# Unveiling the Neurotoxic Effects of Ochratoxin A and Its Impact on Neuroinflammation

**DOI:** 10.3390/toxins17060264

**Published:** 2025-05-23

**Authors:** María Ángeles García-Esparza, Eva María Mateo, José Antonio Robles, Michela Capoferri, Misericordia Jiménez, José Miguel Soria

**Affiliations:** 1 Department of Pharmacy, Cardenal Herrera University—CEU Universities, 46001 Valencia, Spain; 2 Department of Microbiology and Ecology, School of Medicine and Dentistry, University of Valencia, 46001 Valencia, Spain; eva.mateo@uv.es; 3 Department of Biomedical Sciences, Cardenal Herrera University—CEU Universities, 46001 Valencia, Spain; robles.el@uchceu.es; 4 Departament of Animal Production and Animal Health, Veterinary Public Health and Food Science and Tecnnology, Cardenal Herrera University—CEU Universities, 46001 Valencia, Spain; michela.capoferricapoferri@alumnos.uchceu.es; 5 Department of Microbiology and Ecology, University of Valencia, 46100 Valencia, Spain; misericordia.jimenez@uv.es

**Keywords:** inflammation, mycotoxins, nervous system, neurodegeneration, ochratoxin A

## Abstract

Ochratoxin A (OTA), a toxic compound generated by *Aspergillus* and *Penicillium* fungi, is a common contaminant in different food and animal feed sources, thereby posing possible dangers to human well-being. Although OTA is widely recognized for its kidney-damaging properties, new findings have also indicated its potential to harm the nervous system. Current research trends have increasingly examined the part played by environmental poisons, such as mycotoxins, in the development of diseases. This systematic review gathers and assesses the features of OTA along with the insights acquired from studies on its neurotoxicity. This work presents recent research that demonstrates some mechanisms by which OTA crosses the intestinal and blood–brain barriers, penetrating neural structures. In addition, it discusses the effect of OTA on several types of neural cells and its roles in apoptosis, neuroinflammation, and neurogenesis defects, while also determining the effects of antioxidant systems that neutralize the effects of OTA. This paper identifies crucial gaps in the research and highlights the necessity for further in-depth studies into how OTA affects the processes underlying neurodegeneration. Filling these knowledge gaps could provide valuable insights into the neurotoxic potential of OTA and its relevance to neurological disorders.

## 1. Introduction

Mycotoxins represent the toxins with the highest probability of entering the human body owing to shifts in the natural environment or incorrect handling of food items. Such conditions can encourage their presence and dissemination across food crops and their derived products [1] These toxins are secondary metabolites produced by fungi and can be poisonous to vertebrates through ingestion, inhalation, or dermal contact. Their presence in the food chain can arise from fungal infection of crops, either via direct human consumption or following their use as animal feed. Entry into the food supply through animal sources is most probable through meat, milk, or eggs [1]. Crucially, the metabolism of mycotoxins that have been ingested can lead to their accumulation within various organs or tissues [1,2]. Prior to 1985, the Food and Agriculture Organization of the United Nations (FAO) estimated that roughly 25% of world cereal production is contaminated by mycotoxins [3,4]. Furthermore, other foodstuffs, including nuts, spices, fruits, and products derived from them can also be contaminated by these fungal metabolites.

Mycotoxin production in agricultural crops can occur at various points in the food chain, encompassing pre-harvest, harvest, drying, and storage. Importantly, inadequate agricultural and harvesting practices, and improper drying, handling, packaging, storage, and transport conditions all encourage fungal growth, thereby elevating the risk of mycotoxin production. Once food has undergone processing, further mycotoxin production is unlikely provided that food commodities are stored under conditions that prevent fungal contamination and mycotoxin bioproduction, particularly if the water activity of the product is sufficiently low to prevent mold growth and mycotoxin production, with the latter being key to the production of products free from mycotoxins [2].

Epidemiological links between several mycotoxins and a diverse array of health issues, encompassing neurological and developmental effects, have been established in both animals and humans [1,5,6,7]. Mycotoxins are considered the most prevalent food-related external toxin capable of affecting the brain, with some also known to traverse the blood–brain barrier (BBB), thus permitting their accumulation in brain tissues where they disrupt the normal function of the central nervous system (CNS) [8,9,10]. Moreover, the part played by mycotoxins in making individuals more susceptible to the inherent neurotoxicity of nerve diseases is gaining increased recognition among researchers. Of note, the Aflatoxin B1 (AFB1), Deoxynivalenol (DON), T-2 toxin (T-2), OTA, Fumonisin B1 (FB1), and 3-Nitropropionic acid (3-NPA) mycotoxins, which have strong affinity with brain tissues, can enter the systemic circulation both through consumption or skin contact, and some of their metabolites can easily cross the BBB to reach nerve cells via transportation proteins [11,12].

In this current study, we focused on the detrimental impact of mycotoxins in terms of human health, especially in the CNS. To this end, we focused on ochratoxins and in particular, ochratoxin A (OTA), as the main toxin with notable effects on the nervous system.

For this review, a bibliographic search was conducted by using PubMed, Science Direct, and Web of Science. Given the specificity of the toxin and its impact on the nervous system, no time limit has been established for the bibliography provided in this review.

## 2. The Characteristics of Ochratoxin A and Its Relationship with the Nervous System

### 2.1. Origin, Contamination, and Characteristics of Ochratoxin A

**Figure 1:**
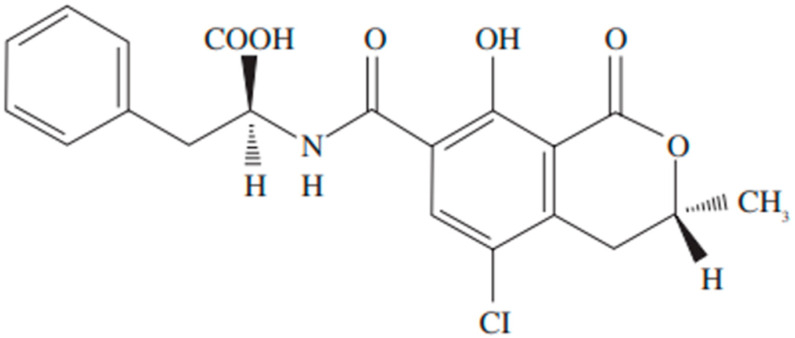
Chemical structure of ochratoxin.

**Figure 2:**
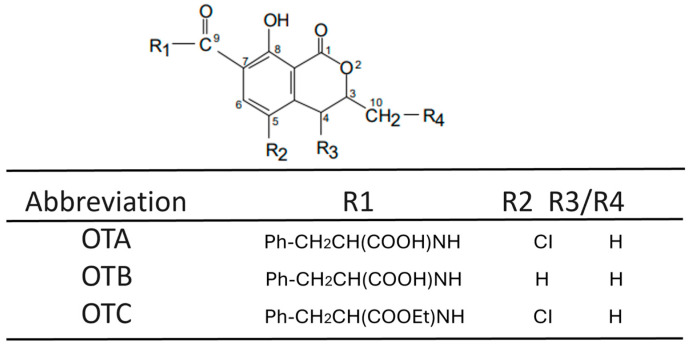
Ochratoxin A analogues.

Many elements such as genetic makeup, typical physiological functions, the external environment, and the quality of food each play a role in human health. Nevertheless, numerous chemical dangers represent significant threats to our health [13], with mycotoxins representing a significant category of such dangers. Humans are exposed to mycotoxins via various routes, such as the respiratory system, skin, and gastrointestinal tract, the latter constituting the primary route for long-term exposure [14]. Mycotoxins can have damaging effects on a range of organs, such as the liver, kidney, and immune system; furthermore, they can induce cancer, disorders of the reproductive system, and other functional impairments. Notably, a growing body of evidence suggests that these toxins may contribute to human neurodegenerative conditions [15,16]. Several studies, both epidemiological and experimental, indicate that certain chemical contaminants present in food can cause harmful effects by targeting the CNS, specifically nerve cells and glial cells [17,18,19]. Ochratoxins, a type of mycotoxin frequently produced by *Aspergillus* and *Penicillium* species as metabolic by-products [20,21], exist in three distinct forms: ochratoxin A, B, and C [22], as illustrated in Figure 1 and Figure 2.

OTA (C_20_H_18_C_l_NO_6_; molecular weight: 403.8) Ochratoxin A has been shown to be one of the most toxic forms compared to the other forms described [23]. It presents as a white, odorless, heat-resistant (with a melting point of 168–173 °C), solid crystalline substance exhibiting limited solubility in water [22]. The production of OTA occurs in numerous common fungal molds and can be detected in cereals, coffee, grapes, and meat. Typically, the reported mean concentration of OTA varies between 0.1 and 100 nanograms per gram of plant-derived foodstuffs [24]. Moreover, derivative products from wheat, oats, barley, rye, maize, rice, millet, sorghum, soybeans, broad beans or other beans, peas, alfalfa, sunflower, or pumpkin seeds, coconut, peanuts, and hay after silage process can exhibit OTA levels ten times higher (ranging from 1 to 100 ng/g) [25]. In human and animal feed derived from animals such as pig blood products, pork meat and edible offal, chicken meat and offal, and dry-cured ham, OTA concentrations are reported to range from 0.1 to 1 ng/g [25,26].

The European Commission has established limits through Commission Regulation (EU) 2023/915. In the past years, regulation for mycotoxins has been changing a lot, and the last regulation was set in 2023 [27]. Thus, The European Commission has established limits for the maximum permissible level of OTA in various food products, including unprocessed cereal grains (5.0 parts per billion), processed cereals (3.0 ppb), and beer and grape juice (2.0 ppb). Furthermore, the Joint FAO/World Health Organization (WHO) Expert Committee on Food Additives (JECFA)—a specialist organization offering scientific guidance to the Codex Alimentarius Commission—has repeatedly considered OTA (in 1991, 1995, 2001, and 2007), recently (2023) establishing a maximum limit of 5 μg/kg in wheat, barley, and rye under the Codex General Standard for Contaminants and Toxins in Food and Feed [27,28].

### 2.2. The Toxicokinetics of Ochratoxin A

The uptake of OTA via the gastrointestinal tract facilitates its entry into the systemic circulation, enabling its subsequent detection in blood and various tissues, particularly in organs exhibiting high metabolic activity such as the kidney, liver, muscle, and fat [29]. During its circulation, OTA exhibits a strong ability to bind with plasma proteins but its elimination half-life is prolonged, ranging from 72 to 120 h in pigs and reaching 840 h (35 days) in humans [30,31]. OTA has also been detected in milk secretions which constitutes a risk for newborns, directly affecting their growth and development [32,33,34]. Both OTA and its metabolites are excreted via the kidneys and the hepatobiliary system.

**Figure 3:**
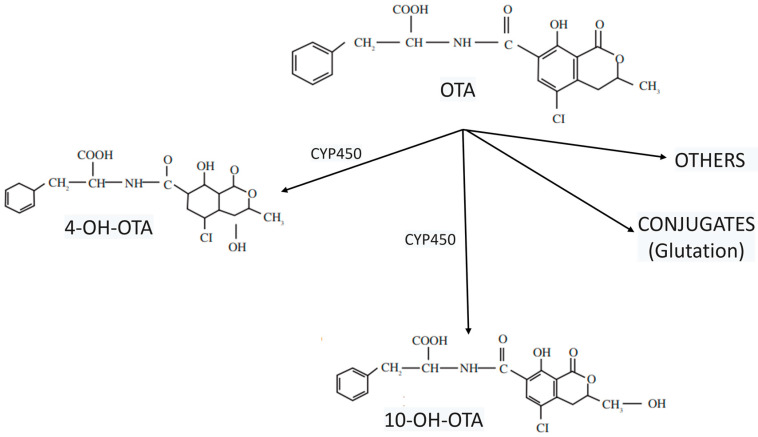
Hydroxylated derivatives (4-OH-OTA and 10-OH-OTA) from OTA and conjugation products with glutathione.

The metabolism of OTA is varied and significantly dependent on the species. In contrast to other species, humans possess a comparatively limited metabolic capacity for OTA, with the most significant metabolic pathways including amide or lactone moiety hydrolysis to form ochratoxin alpha (OTα) or open lactone OTA, respectively, hydroxylation to form hydroxylated derivatives (4-OH-OTA and 10-OH-OTA), and glutathione (GSH) conjugation reactions (Figure 3). All the metabolic products resulting from these reactions exhibit lower toxicity compared to OTA, with the exception of open lactone OTA [35]. The main hepatic metabolites appear to be the epimers (4R and 4S)-OH-OTA, formed by the cytochrome P450 (CYP450) system [36], with CYP450 enzymes also generate hydroxylated metabolites and other less prevalent metabolites, including OTA-quinones.

Nevertheless, contradictory findings have been reported concerning the involvement of biotransformation products in the harmful effects of OTA, with several studies suggesting that different biotransformation pathways contribute to OTA-induced cytotoxicity [36]. Given that numerous studies are beginning to reveal the different implications of OTA derivatives in human health, it is important to understand the kinetics and characteristics of these different metabolites. Interestingly, some data propose that simultaneous exposure to structurally related mycotoxins, which induce DNA damage through entirely distinct mechanisms, may substantially elevate the risk of cancer in humans who consume mold-contaminated foods [37].

## 3. Ochratoxin Crosses the Gastrointestinal and Blood–Brain Barrier

The gut lining constitutes a vital barrier composed of interconnected elements and serves as the primary defense mechanism against infection by impeding the movement of potentially damaging toxins, bacteria, and viruses from the intestinal lumen into the circulation. The function of this barrier relies on the dynamic interplay between the luminal microbiota, epithelial cells, and immune cells located in the lamina propria (LP). A single layer of epithelial cells covers the intestinal wall and is crucial for maintaining this physical barrier [38]. Within this context, the gut lining is composed of four principal intestinal cell types: absorptive enterocytes, which form the majority of the cellular components; protective mucus-producing goblet cells; Paneth cells, which generate antimicrobial peptides (AMPs); and hormone-secreting enteroendocrine cells [39].

Epithelial cells exhibit connections to one another in the basolateral-to-apical direction by way of interconnected protein contacts, known as apical junctional complexes. These complexes include desmosomes, adherens junctions (AJs), and tight junctions (TJs) [40]. Molecules traverse this gut lining via transcellular and paracellular pathways, with the latter being controlled by TJs. Consequently, TJs selectively regulate the flow of nutrients and stimuli and are regarded as the principal factors determining intestinal paracellular permeability [16,41,42].

**Figure 4:**
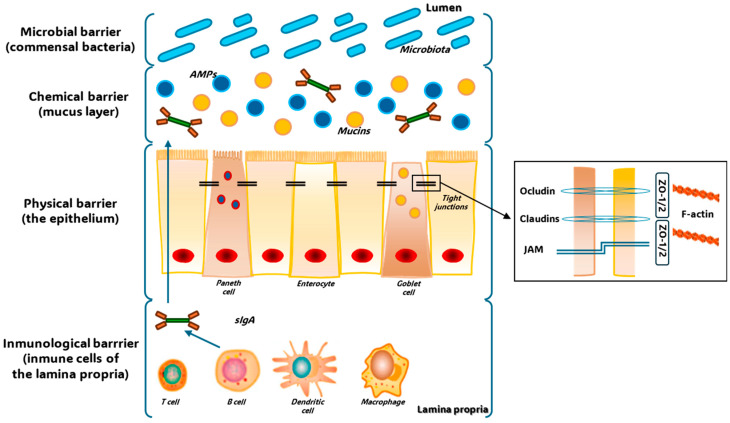
The maintenance of normal gut equilibrium relies on a four-tiered defense system against external challenges. This system incorporates a physical component (a single layer of epithelial cells with selective permeability), a chemical component (a mucus layer containing mucins and antimicrobial peptides, released by goblet and Paneth cells, respectively), an immunological component (immune cells residing within the lamina propria and secreted immune signaling molecules like cytokines and secretory immunoglobulin A [sIgA]), and a microbial component (the community of commensal bacteria within the intestinal lumen). Adjacent epithelial cells are joined by tight junctions, complex structures of transmembrane proteins such as junctional adhesion molecules (JAMs), claudins, and occludin, which are anchored to the actin cytoskeleton via zonula occludens (ZO) proteins (adapted from [45]).

As represented in Figure 4, the intestine is protected from external stimuli in four ways: through the formation of a physical barrier (a solitary layer of partially permeable epithelial cells); and a chemical barrier (a mucus layer made up of mucins and antimicrobial peptides secreted by goblet cells and Paneth cells, respectively); an immunological barrier (through immune cells in the lamina propria (LP) and secreted immune mediators such as cytokines and secretory immunoglobulin A); and a microbial barrier (commensal bacteria present in the intestinal lumen). In this model, adjacent epithelial cells are connected by TJs, multiprotein complexes that link adjacent epithelial cells near their apical edge. These complexes are comprised of various transmembrane proteins, including junctional adhesion molecules (JAMs), claudins, and occludin, all of which are linked to the actin cytoskeleton via zonula occludens (ZO) proteins [43,44,45]. Where the gut barrier integrity is poor there is a heightened risk of systemic infection and nutritional deficiency [46,47].

TJs close off the luminal extremity of the intercellular space and restrict transport via this paracellular pathway to only relatively small water-soluble molecules; their permeability alters in response to various physiological and pathophysiological stimuli, including illness and injury [48]. Moreover, it has also become clear that the integrity of the gut barrier is dependent on the presence of micronutrients within the gut. Thus, the gut can respond to signals generated by the presence of the different foods it processes. However, the particular dietary constituents required to maintain the gut barrier remain unidentified and the processes through which alterations in junctional permeability arise are not widely understood. Consequently, within this context, OTA presents a significant risk to the health of both humans and animals. Indeed, despite OTA being nephrotoxic, immunotoxic, and carcinogenic, its main site of action is the kidneys [49], its initial interaction following ingestion occurs with the gut lining [50].

To understand the effect of OTA on the intestinal barrier at the molecular and cellular levels, studies using Caco-2 cells (a well-established intestinal model cell line [51] have indicated that this toxin does not destroy TJ complexes but rather, ‘loosens’ them, thereby making the transit of smaller molecules through the paracellular pathway easier [52]. Consequently, it has been proposed that OTA specifically impacts claudins rather than inducing substantial alterations in cells. One study demonstrated that exposing both the apical and basolateral surfaces to OTA led to increased gut permeability, reduced the expression of TJ proteins, and altered the TJ protein distribution pattern [53]. These effects on TJ protein localization were confirmed through transmission electron microscopy [54]. Therefore, in vitro treatment with OTA resulted in heightened cell permeability and disrupted microvilli and TJ proteins in various cell culture systems [53]. Moreover, the detrimental effects of OTA on the structure of the intestine have also been documented in various in vivo models [55]. All these observations indicate that when the intestine experiences short-term exposure to low concentrations of mycotoxins, inherent cellular mechanisms maintain the integrity of the physical gut barrier. However, prolonged damage that surpasses the capacity for self-regulation disrupts intestinal epithelial cells and TJ proteins, resulting in barrier compromise. Similarly, extended exposure of animals to low mycotoxin doses can also negatively impact gut health.

Once OTA traverses the gut barrier, it enters the bloodstream and therein, its route to the nervous system requires that it crosses the BBB, a permeability barrier situated between the bloodstream and cerebral endothelial cells. In most vertebrates, the BBB controls the interchange of compounds between the blood circulation and the brain. Furthermore, it protects neurons from changes in osmotic pressure and xenobiotics or potentially harmful endogenous compounds in the bloodstream. Three types of cells, which can also be referred to as a neurovascular unit, form the BBB: cerebral microvessels are comprised by endothelial cells that surround blood capillaries. Their primary role constitutes the essential foundation of the barrier by governing the entry of nutrients and the exit of potentially harmful substances [56]. A basal lamina envelops this layer of endothelial cells, also housing pericytes within its structure. Extensions from astrocytes, known as end feet, are positioned on this basal lamina, almost completely encompassing it. Both pericytes and astrocytes perform a range of regulatory roles, influencing the impermeability of the endothelial barrier and the expression of proteins [57,58].

To uphold its principal barrier functions, its operation can be categorized into three characteristics. Firstly, the physical barrier is formed by TJs, which close the gaps between the endothelial cells and minimize paracellular diffusion. The transmembrane proteins that constitute TJs, such as occludins or claudins, are linked to the actin cytoskeleton, forming the physical barrier and accounting for the high trans-endothelial electrical resistance (above 1000 Ω·cm²) of the BBB [59]. Different dose-dependent studies both in vitro and in vivo have shown that ingestion of OTA generates brain damage at different levels [5,8,60,61,62,63,64]. Indeed, the presence of OTA in the brain seems to suggest that it crosses the BBB where it then settles in different neural structures [65].

**Figure 5:**
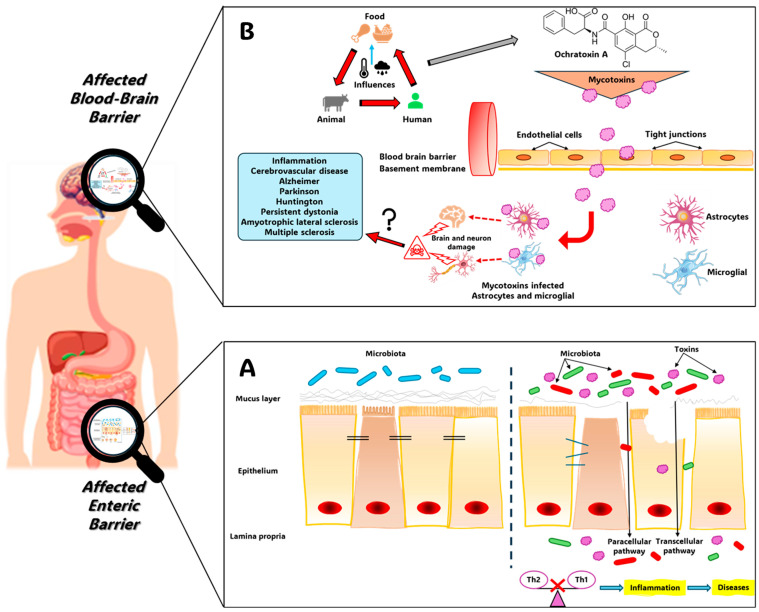
(**A**): Mycotoxins exert detrimental effects on the gut barrier through several key mechanisms. These include (i) elevated permeability, affecting both paracellular and transcellular transport pathways, which arises from damage to epithelial cells and tight junctions, and (ii) a reduction in the thickness of the mucus layer. This compromised gut barrier allows for the entry of foreign substances of varying molecular sizes and the movement of bacteria across the intestinal lining, ultimately contributing to a disruption of the balance in inflammatory conditions (adapted from [45]). (**B**): Mycotoxins originating from food sources can induce damage to neurons and the brain by affecting astrocytes and microglia. Mycotoxins, such as ochratoxin A (OTA), primarily enter the body via contaminated crops and foods derived from animals (including meat, eggs, milk, sugarcane, and edible offal). Both mycotoxins and their breakdown products can readily cross the blood–brain barrier (BBB) and affect astrocytes and microglia, potentially leading to neuronal and brain damage. The question of whether mycotoxins contribute to neurodegenerative diseases, such as Alzheimer’s, Parkinson’s, Huntington’s, and amyotrophic lateral sclerosis, requires further investigation (adapted from [66]).

An in vitro investigation into the effects of OTA on the BBB has employed Porcine Brain Capillary Endothelial Cells (pBCEC) to show that 10 µM of OTA was cytotoxic while 1 µM concentration weakened the barrier. In contrast, other toxins like OTα, citrinin (CIT), or dihydrogencitrate (DHCIT) did not compromise BBB integrity or induce cytotoxic effects [65]. Despite their molecular characteristics and significant fat solubility, the rates at which these four mycotoxins crossed the barrier, and their permeability were notably less than anticipated. This unexpected outcome can be explained by the action of efflux transport proteins, which actively moved substances back into the bloodstream or the apical compartment within this experimental setup [65], which was the strongest contributor to OTA excretion from renal tissues. Figure 5 shows the negative effects of OTA on the intestinal barrier and BBB. Although this knowledge is based on recently published studies, further work will still be required to reveal the mechanisms by which OTA is able to cross the BBB.

## 4. Cellular Effects of Ochratoxin A: An Insight into the Nervous System

The differing toxic effects of OTA on humans and livestock have been recognized for a considerable time. However, in recent years, health agencies have allocated increased resources to researching food contamination by mycotoxins, especially by aflatoxins, fumonisin, OTA, patulin, trichothecenes, and zearalenone. The current European legislation CE n. 1881/2006 and n. 1126/2007 establish the maximum levels of mycotoxins allowed in human food but nevertheless, regulations regarding certain products still remain unclear [67]. As already mentioned, these toxins have a range of harmful effects on human health; OTA is nephrotoxic, hepatotoxic, teratogenic, immunotoxic, neurotoxic, and tumorigenic [5,9,19,60,61,68].

Recent studies focused on the neurotoxicity of OTA have suggested it builds up in the brain resulting in oxidative stress and DNA damage in several brain areas and nerve cell populations [5,61,69,70]. In fact, the neurotoxicity of OTA has been shown to be most evident in the cerebellum, ventral mesencephalon, hippocampus, and striatum brain regions [8,64]. Indeed, the detrimental effect of OTA on the CNS is garnering increasing attention due to several laboratory and animal studies in which OTA exposure was linked to neurological and neurobehavioral impairments [60,61,70]. Nevertheless, research into the effects of acute or chronic OTA exposure on the CNS remains limited, despite indications that developing nervous tissue is particularly vulnerable to its damaging effects [71,72,73].

Different studies have indicated that OTA hinders the creation of new nerve cells in the hippocampus, both in living organisms and in laboratory settings, and this effect may be linked to problems with learning and memory [8,61,69,70]. In this context, exposure to low levels of OTA may produce delayed nerve-damaging effects, which could subsequently play a part in the development of neurodegenerative conditions [8,73]. These toxicological effects on the hippocampus add to other findings demonstrating the deleterious effects of OTA on cell proliferation and differentiation processes in fetal midbrain cell cultures [74], the developing brain [75], and nerve stem or progenitor cells in the adult hippocampus [61,70]. Furthermore, the harmful effect of OTA on the creation of new nerve cells in the hippocampal dental gyrus of adult mouse brains has also been documented. Indeed, intraperitoneal injection of male mice with 1–6 doses of OTA (3.5 mg/kg body weight) over three days led to a notable dose-dependent reduction in the length and number of astrocyte branches, glial fibrillary acidic protein (GFAP) expression, and quantity of astrocytes and young and mature neurons [61].

**Figure 6:**
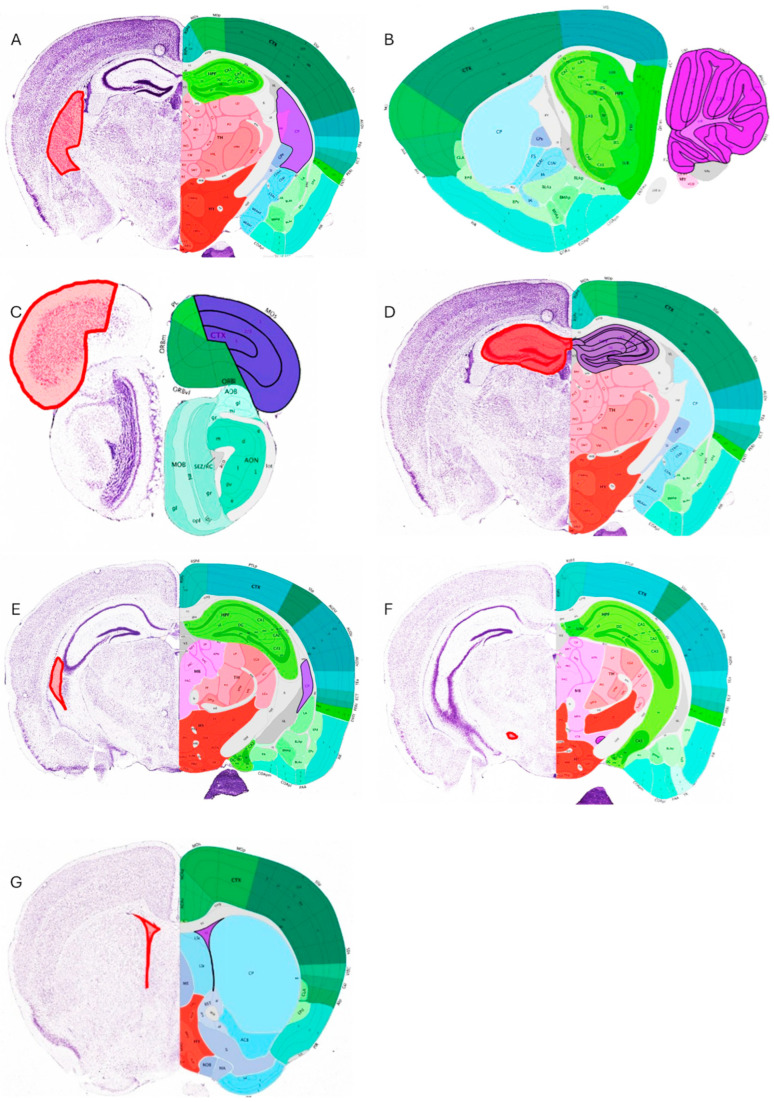
Brain mouse affected areas. (**A**), caudoputamen, Yoon et al. (2009) [68] and Sas (2007) [63]; (**B**), cerebellum, (**C**), cortex frontalis, Sas (2007) [63]; (**D**), hippocampus, Mateo et al. (2022) [61], Yoon et al. (2009) [68] and Sava et al. (2007) [69]; (**E**), striatum, Sas (2007) [63]; (**F**), substantia nigra, Sas (2007) [63]; (**G**), subventricular zone (SVZ), Paradells et al. (2015) [5]; Sas (2007) [63]; Modified from Allen Mouse Brain Atlas [dataset], Allen Institute for Brain Science (2011). The red line shows the affected anatomical structure.

Moreover, there have been reports on the effects of OTA exposure on other neurogenic parts of the brain such as the subventricular zone (SVZ). The latter has been investigated for its importance in adult neurogenesis and the replacement of neural populations during adult life. The laboratory and animal effects of OTA exposure on the SVZ and neural precursors taken from this neurogenic niche suggest that OTA could harm the survival, multiplication, and specialization of both developing and adult SVZ cells, depending on the amount of exposure [5]. Figure 6 describes the locations affected by OTA in rodent brains.

As noted earlier, a strong link exists between OTA, mutagenicity, carcinogenicity, and genotoxicity, although the precise processes involved are not yet clear. Reports on the genotoxic effects of OTA indicate that it results in the formation of DNA adducts, inhibits protein synthesis, perturbs cellular energy production, initiates oxidative stress, induces apoptosis, alters mitosis, causes cell cycle arrest, and interferes with cytokine pathways. All these processes are linked with kidney and liver damage, teratotoxicity, immunological toxicity, and neurotoxicity. Some studies have also reported a connection between OTA and mental health disorders. In this context, administering OTA activates various pathways including p38 mitogen-activated protein kinases (MAPK) and Jun N-terminal Kinases (JNK), causes Extracellular-Signal-Regulated Kinase (ERK) dysfunction, disrupts brain-derived neurotrophic factor (BDNF), is related to Tyrosine hydroxylase (TH) overexpression, and caspase-3 and 9 activation, as well as ERK-1/2 phosphorylation which ultimately leads to the progression of Alzheimer’s disease (AD) [76].

Furthermore, perhaps because of the range of brain locations affected by OTA, different studies seem to show that it is involved in different psychiatric disorders, demonstrating a relationship between mycotoxins and the development of neuropsychiatric symptoms including brain fog, cognitive impairment, and headaches [77,78]. Research has also demonstrated that sub-chronic oral exposure to OTA elicited pathological phenotypes in mice like those observed in Parkinson’s disease (PD) [73,79,80]. Likewise, various studies have outlined a potential role played by OTA in the disease processes of autism through epigenetic processes [81]. In this context, the authors proposed that a gene–environment interaction might trigger autism spectrum disorder, with the epigenetic neurotoxic effects being triggered by OTA in children with a genetic predisposition [81,82]. The mechanisms responsible for neural tissue toxicity remain unclear, but studies in organs and tissues outside the nervous system have shown that it has a variety of functions, with its processes including the hindering of protein creation, mitochondrial damage, oxidative stress, and DNA damage.

## 5. Ochratoxin A, Mitochondrial Impairment, and Oxidative Stress: The Development of Neurological Diseases with Fatal Outcomes

Numerous investigations have shown that oxidative stress is a key factor in the toxicity of OTA [19,68,83,84,85,86,87]. Oxidative stress indicates an imbalance between the widespread presence of reactive oxygen species (ROS) and the capacity of a living system to readily neutralize reactive intermediates or repair the resulting damage. Disruptions in the normal redox balance of cells can lead to toxic effects through the creation of peroxides and free radicals that harm all cell constituents, including proteins, fats, and DNA [84,88,89]. The brain is particularly susceptible to oxidative stress due to its high metabolic rate and oxygen usage, making it a significant element in the development of many nerve-related disorders.

Excess ROS, encompassing molecules such as superoxide and hydroxyl radicals and hydrogen peroxide (H_2_O_2_), can harm vital biomolecules, setting off a sequence of events that leads to cellular malfunction and, ultimately, to nerve cell degeneration. Consequently, a range of nerve-related illnesses including AD, PD, amyotrophic lateral sclerosis (ALS), Huntington’s disease (HD), multiple sclerosis (MS), and ischemic stroke, are linked to oxidative stress [89,90]. Given this, neuroprotection is a proactive approach to securing the nervous system, encompassing the brain, spinal cord, and peripheral nerves, by preventing or limiting damage to nerve cells and other components; it mainly defends the CNS against injury from acute and progressive nerve degeneration conditions.

Oxidative stress affects cells via mitochondria, which generate 90% of cellular energy as adenosine triphosphate (ATP) and which also regulate calcium metabolism and regulate heat production. Poorly functioning mitochondria produce elevated levels of ROS and reactive nitrogen species (RNS) that, as previously stated, lead to the oxidative damage of biomolecules. The ROS generated by mitochondria includes superoxide anions, H_2_O_2_, and hydroxyl radicals, which are managed by enzymes including Mn-SOD and Cu/Zn-SOD. Peroxisomes, which contain catalases to decompose H_2_O_2_, also contribute to the production of ROS. Antioxidant systems like glutathione S transferase (GST) and thioredoxin (Trx) neutralize excessive ROS and RNS but nonetheless, persistent oxidative stress still contributes to aging and cancer [91,92].

Moreover, the generation of nitric oxide (NO) is a component of oxidative stress, stemming from inadequate equilibrium in the regulation of ROS and RNS. NO has a wide variety of functions including in nerve signaling and blood pressure control, but too much NO can harm cells [93], influence apoptosis, and cause inflammation. Moreover, NO can cause the release of cytochrome c from mitochondria, activating the caspase-dependent cell death pathway and binding to cytochrome c oxidase, leading to mitochondrial dysfunction. NO synthesis from L-arginine by nitric oxide synthases (NOS) involves NOS isoforms, including nNOS, eNOS, iNOS, and mtNOS [94]. When nitric oxide (NO) interacts with superoxide within mitochondria, it forms peroxynitrite, a substance implicated in the development of illnesses like stroke, heart disease, diabetes, inflammation, cancer, nerve degeneration conditions, and the aging process [94]. In this way, oxidative stress plays a key part in the disease processes of dementia, with studies showing links between oxidative stress markers like malondialdehyde (MDA), glutathione peroxidase (GSH-Px), and protein carbonyls (PC) and cognitive decline, particularly in older individuals [94].

In turn, mitochondrial impairment may be one of the major mechanisms of OTA toxicity. Mitochondria represent the main location within cells for oxidative phosphorylation and aerobic respiration, and they also control the production of ROS and the natural occurrence of apoptosis [95,96]. In addition, as already mentioned, poorly functioning mitochondria can trigger oxidative stress, which ultimately leads to cell death period. In this way, oxidative stress and apoptosis caused by OTA in human liver cancer cells might be explained by a loss of mitochondrial membrane potential (MMP) [97]. In vitro experiments examining how OTA affects the membrane structure of rat liver mitochondria it has been observed that OTA reduced the respiratory control of the isolated mitochondria in a dose-dependent manner, inhibiting their electron transfer functions [98].

Another recent study showed that exposure to OTA considerably lowered MMP in neural cells and, in particular, retinal ganglion cells (RGC-5) [99]. These researchers observed that when RGC-5 cells were exposed to OTA, their mitochondria became shorter, with fewer elongated and more spherical ones being present, indicating that this toxin might lead to mitochondrial rupture and thus dysfunction. Moreover, a comparable investigation has also suggested that H_2_O_2_ can cause a reduction in MMP, triggering apoptosis in RGC-5 cells [100]. Furthermore, earlier work on glial cells indicated that OTA reduced cell multiplication, leading to G0/G1 cell cycle arrest. This was linked to a decrease in the expression of *CCND1*, *CCNE1*, *CDK4*, and *MYC*. OTA also triggered cell death in NHA-SV40LT cells by causing a loss of MMP and an increase in *BAX* and *TP53* levels. Consequently, OTA appears to cause neurotoxicity in human astrocytes by hindering cell growth and initiating cell death through mitochondrial pathways [78].

While neurons and astrocytes are fundamental brain cells affected by the neurotoxic effects of OTA, recent studies have also revealed it has similar effects on microglia [101]. Microglia are the principal neuroimmune cell type and perform three crucial roles: they function as sentinels that continuously detect alterations in their surroundings; housekeepers that maintain a healthy nerve environment and normal function; and neuroprotective defenders that respond to said changes. Microglia are involved in both neuroinflammation and neurodegeneration [102], with the start or exacerbation of the latter resulting from imbalances in microglial function [102,103,104].

## 6. The Role of Ochratoxin A in Neuroinflammation

The part played by nerve inflammation as an initiator of nerve cell degeneration is clearly recognized, with a proven connection between neuronal inflammation and nerve cell loss in several nerve degeneration conditions [105]. Neuroinflammation is a defense mechanism that initially protects the brain by removing or inhibiting a wide range of pathogens. The inflammatory response of the brain is triggered by the activation of microglial cells and astrocytes in response to various types of CNS injury, pathogens, or even neurotoxic insults. The outcome of neuroinflammation is determined by cellular interactions, inflammatory mediators, and trophic and/or cytotoxic signals. It also hinges on various other elements such as how strong and how long the harmful event lasts, the degree of initial nerve cell damage and glial response, and how developed the brain is. Consequently, depending on the situation, the neuroinflammatory response might encourage nerve protection, regrowth, or nerve cell degeneration [106]. In the context of the above, glial cell response is seen as the key occurrence in brain inflammation and thus, has been employed as an initial indicator of nerve damage.

Neuroinflammation manifests as increased levels of pro-inflammatory cytokines, activation of macrophages (microglia), the movement of white blood cells from the body into the nervous system, and damage to nerve tissue [107]. Microglia are cells specialized in day-to-day homeostatic functions, including correcting neuronal hyperactivity [103], which participates in neural-circuit remodeling [108], neurogenesis [109], and control the composition of the CNS milieu [110]. In response to stimuli such as pathogens, microglia secrete inflammation-promoting signaling molecules including interleukin (IL)-1b, IL-16, tumor necrosis factor (TNF)-α, and chemokines. In turn, they can mediate the activation of pro-inflammatory astrocytes and consequently initiate a secondary inflammatory response. Although nerve inflammation serves as a nerve-protecting process, it is important to remember that when sustained, it can induce neurotoxicity and is related to neurodegeneration [102]. Moreover, OTA exposure induces an inflammatory response in astrocytes and microglial cells which promotes morphological changes and increased expression of cytokines and stress proteins [60,111,112].

### 6.1. The Astrocyte-Specific Effects of Ochratoxin A

Astrocyte reactivity can be tracked by the increased production of glial fibrillary acidic protein (GFAP) [113], a type III intermediate filament protein central to inflammatory processes that might also have a part in altering nerve cell survival or degeneration. Elevated GFAP production as an indicator of astrocyte response (also termed astrocytosis, astrogliosis, or the gliotic response), can be seen at both the mRNA and protein levels [114,115]. In aggregate cultures, the presence of OTA decreased GFAP immunoreactivity [111] and GFAP expression, with GFAP mRNA and protein downregulation occurring as soon as 24 h post-treatment. OTA simultaneously caused the upregulation of vimentin, another intermediate filament protein, which can be co-expressed with GFAP in certain reactive astrocyte populations. Intriguingly, in brain cell cultures grown without serum and allowed to clump together, a reduction in GFAP production and an increase in vimentin mRNA levels were more noticeable in cells from older animals following treatment with very low concentrations of OTA below the cytotoxic level (10 nM and 20 nM) [112].

The OTA-induced modulation of astrocyte response in aggregating brain cell cultures suggests that cytoskeletal changes may be central to this process. In support of this notion, cyclic AMP, which enhances *GFAP* expression, can partially counteract the decrease in *GFAP* and inflammatory marker upregulation brought about by OTA [112]. In another experiment, histotypic rat brain cell cultures were simultaneously exposed to extremely low concentrations of OTA (2 nmol or 10 nmol) and two astrocyte-specific proteins, metallothioneins I and II (MTI/MTII, at 25 ng/mL each) for 10 days. At the 10 nM OTA exposure level, the authors reported a rapid decrease in MTI, MTII, and GFAP after 48 h up until day 10, suggesting that these astrocytic proteins could have a significant part to play in astrocyte–microglial communication and the upregulation of microglial activity [114,115]. In vivo, OTA produced dose-dependent morphological changes in astrocytes and in GFAP markers in the hippocampus of male C57BL/6 mice, with a cumulative 60% decrease in GFAP-positive cells after six doses of 3.5 mg OTA/kg administered intraperitoneally [61].

### 6.2. Microglia Activation and the Effects of Ochratoxin A

OTA is thought to cross the BBB, but the mechanism of microglia activation therein still remains unknown, although it has been suggested that OTA-mediated neurotoxicity happens due to the activation of MAPK activation [116,117]. In mammals, MAPKs include c-Jun NH2-terminal kinase (JNK), p38 MAPK, and ERK. These enzymes are serine–threonine protein kinases that regulate various cellular activities including proliferation, differentiation, apoptosis and survival, inflammation, and innate immunity. Of note, compromised MAPK signaling pathways contribute to the pathology of several human diseases including cancer and neurodegenerative disorders such as AD, PD, and ALS. In particular, JNK and p38 MAPK signaling are activated by oxidative, genotoxic, and osmotic cell stress as well as by proinflammatory cytokines such as TNF-α and IL-1β. The p38 MAPK pathway contributes to neuroinflammation mediated by glial cells, including microglia and astrocytes [118].

To elucidate if OTA activates MAPKs, particularly the ERK, JNK, and p38 pathways, a series of experiments were conducted in immortalized mouse microglial cells (BV-2 cells) with ERK, p38 MAPK, or JNK inhibitors at different OTA concentrations [50, 250, and 500 nM) over 24 h. The results showed that OTA activated microglia through ERK and p38 MAPK but not JNK. Furthermore, MAPK signaling has a vital part in microglial cell activation by regulating the production of inflammation-promoting agents [119]. At the neuronal level, experiments with BV-2 cells have also shown that OTA directly affects the activation of the neuroinflammatory response in a dose-dependent manner, increasing IL-6 and TNF-α levels after only 24 h of exposure at a concentration of 50–2000 nM [119].

Similarly, recent studies in immortalized human microglia SV40 cells cultured at different OTA concentrations (0.1 nM–100 nM) showed that this toxin can stimulate microglia to release of IL-1β, IL-18, and CXCL8 in a dose-dependent manner [101]. An increase in IL-6 and TNF-α has also been observed in vitro studies with the SH-SY5Y undifferentiated human neuroblastoma cell line when exposed to OTA (3.1–12.5 μM) for 24–48 h, with a dose-dependent increase in the TNF-α response after 48 h [120], as also seen in 3D rat whole-brain aggregate cultures [120,121].

### 6.3. Oligodendrocyte Markers Affected by Ochratoxin A Exposure

Oligodendrocytes, which form the myelin sheath around nerve fibers, develop from oligodendrocyte precursor cells through tightly orchestrated processes of migration, proliferation, and differentiation [122]. They provide electric insulation for CNS axons by creating the myelin sheath that enables saltatory conduction of nerve impulses, ensuring proper neuronal function. Given that axon myelination is an energy-consuming process associated with a high metabolic turnover, oligodendrocytes are especially vulnerable to cytotoxic and excitotoxic factors [123].

## 7. From Ochratoxin A-Induced Neuroinflammation to the Development of Neurodegenerative Diseases

Neuroinflammation is the initial response by activated glial cells and neurons to shield the brain from harm, infections by microbes or blood poisoning, or exposure to toxins. However, inflammatory molecules secreted by microglia can weaken the BBB, permitting the entry of immune cells from the body which can activate and harm glial cells and neurons, thereby contributing to nerve degeneration processes [124]. Thus, given the role of OTA in neuroinflammation, it is important to take a neurological perspective to evaluate how exposure to this toxin at different doses can affect neuronal viability and survival and lead to long-term neurodegeneration diseases. In this sense, astrocytes are crucial for neuron survival because they respond to and reabsorb neurotransmitter overload to end synaptic processes. The glutamate–glutamine cycle is the main metabolic pathway involved in coupling astrocytes and neurons.

Glutamate is the primary excitatory neurotransmitter in the mammalian CNS and can be neurotoxic, leading to nerve cell death [125]. Astrocytes take in and neutralize glutamate using sodium-dependent excitatory amino acid transporters (EAATs) such as excitatory amino acid transporter 1 (EAAT1) or glutamate-aspartate transporter 1 (GLAST) and EAAT2 or glutamate transporter 1 (GLT1). Next, it is transformed into glutamine by astrocytes via glutamine synthetase (GS) and is extracellularly released to be reabsorbed by neurons. Thus, the clearance of excessive glutamate levels by astrocytes prevents excitotoxicity, functional impairment, and neuronal death. More recent findings have indicated that OTA influences how EAATs are positioned within the outer layer of primary astrocytes, thereby reducing GLT1 production and leading to an inability to effectively capture L-glutamate within the nervous system [126]. Indeed, mice subcutaneously injected with OTA for 2 weeks (cumulative dose of 8 mg/kg using Alzet osmotic minipumps) exhibited striatal dopaminergic depletion [73]. Similarly, male Balb/C albino mice orally exposed to OTA (0.21 and 0.5 mg/kg) showed motor impairment and dopaminergic system dysfunction, even up to 6 months after exposure [79].

Nevertheless, further study of gene expression associated with neuronal survival and viability will be important to elucidate the effect of OTA and its implications in neurodegenerative diseases. Several genes such as cellular tumor protein (*p53*) and apoptosis regulator BAX (*BAX*) are upregulated in normal human astrocyte cells treated with OTA (0.5, 1, and 2 μM) [78]. In addition, dysfunctions of *BDNF*, which plays an important role in brain functions such as plasticity, memory, and cognition, have been associated with neurodegenerative diseases and other psychiatric disorders [127]. Furthermore, microtubule-associated protein tau (*MAPT*) and tubulin polymerization-promoting protein (*TPPP*), which both have microtubule functions, have also been connected to nerve-degenerating conditions [128]. The impact of OTA on neuronal survival and viability, specifically in the expression of *MAPT*, *BAX*, *P53*, *BDNF*, and *TPPP* as measured by RT-qPCR, was investigated in SH-SY5Y cells exposed to OTA (2 fM, 20 fM, 2 pM, or 1 µM) over 1, 2, or 11 days. *BDNF* expression significantly reduced after 11 days at 2 pM and *P53*, *BAX*, and *MAPT* expression reduced after 1 and 2 days at 1 µM, suggesting that *BDNF* expression was negatively affected at significantly higher doses, which could have implications for long-term neuronal health [129].

## 8. Role of Antioxidants in Neuroprotection

The CNS is highly susceptible to oxidative damage because of its elevated oxygen consumption, high lipid content, and relatively limited endogenous antioxidant defenses [130]. Additionally, given that the human brain contains upwards of 86 billion neurons and 250–300 billion glial cells, it consumes over 20% of resting oxygen levels, thereby amplifying its vulnerability to oxidative stress [131]. This stress plays a key role in neuroinflammation given that the excessive production of ROS and RNS further exacerbates neuronal damage [132]. Antioxidants are essential in mitigating the harmful effects of ROS and RNS to counteract oxidative stress and modulate the inflammatory response. By scavenging free radicals, antioxidants help preserve cellular integrity, protect neuronal function, and prevent the amplification of neuroinflammation [133].

The cellular antioxidant defense system, which shields tissues from oxidative injury, comprises both endogenous and exogenous antioxidants capable of neutralizing a wide range of harmful molecules. These defenses are categorized into enzymatic and non-enzymatic groups, including oxidative enzyme inhibitors, cofactors for antioxidant enzymes, ROS/RNS scavengers, and transition metal chelators, all working synergistically to reduce cellular stress and maintain homeostasis [134]. Among the key endogenous antioxidants, GSH is a central player that acts as a primary defense mechanism against ROS and RNS inside neurons. By directly neutralizing harmful free radicals, GSH helps maintain the redox balance and prevents oxidative damage to cellular components such as proteins, lipids, and DNA [135].

In this context, mycotoxins such as OTA have been shown to contribute to oxidative stress in the CNS by depleting intracellular GSH levels, thus exacerbating neuronal damage. Supplementation with GSH precursors such as N-acetylcysteine (NAC) can restore GSH levels, reducing lipid peroxidation, protein oxidation, and mitochondrial damage. Furthermore, GSH is crucial in detoxifying OTA through conjugation reactions facilitated by GSTs, thereby promoting OTA elimination and mitigating its toxic effects on neuronal cells [136]. Additionally, antioxidant enzymes such as superoxide dismutase (SOD) and catalase are crucial because they catalyze the conversion of superoxide radicals and H_2_O_2_ into less harmful molecules to prevent further oxidative damage [137]. Together, these endogenous antioxidants work in concert to protect neurons from the damaging effects of oxidative stress and to support cellular homeostasis.

Exogenous antioxidants, including polyphenols, α-tocopherol (vitamin E), ascorbic acid (vitamin C), and coenzyme Q10 (CoQ10) also significantly contribute to neuroprotection by helping to maintain neuronal health and reduce oxidative damage. Polyphenols represent the largest group of phytochemicals, with many of these compounds found in plant-based foods. Abundant in fruits, vegetables, and tea, polyphenols exhibit potent antioxidants and anti-inflammatory effects. These compounds play a crucial role in reducing oxidative damage and modulating the signaling pathways involved in inflammation. Hence, through their ability to interfere with biochemical pathways that regulate the inflammatory response and protect against oxidative stress, polyphenols contribute to the prevention of various chronic diseases associated with inflammation and cellular aging, including AD and PD [138].

In turn, vitamin E, a potent lipid-soluble antioxidant, integrates into cellular membranes, providing protection against lipid peroxidation and preventing subsequent damage to neuronal structures. As a critical component of cellular defense mechanisms, Vitamin E neutralizes ROS and RNS in lipid-rich environments, thereby safeguarding the integrity of cellular membranes against oxidative damage [139]. In the context of neurodegenerative diseases, its role in preserving the fluidity and functionality of neuronal membranes is pivotal, particularly in mitigating processes such as neuroinflammation, mitochondrial dysfunction, and synaptic loss. Indeed, given its multifaceted protective mechanisms, vitamin E has also been investigated for its potential therapeutic applications in the prevention and management of neurodegenerative conditions such as AD and PD [140].

Vitamin C is a water-soluble antioxidant renowned for its ability to neutralize ROS, thereby safeguarding cellular components from oxidative damage. Beyond its direct antioxidative action, vitamin C contributes to the regeneration of other antioxidants, notably vitamin E, enhancing the overall antioxidant defense system. This synergistic interaction ensures a robust protective mechanism against oxidative stress and hence, provides protection against various neurodegenerative disorders [141]. Furthermore, vitamin C serves as a cofactor for enzymes involved in the biosynthesis of neurotransmitters and collagen, underscoring its multifaceted role in maintaining neuronal health and structural integrity [138].

CoQ10 plays a crucial role in mitochondrial bioenergetics, acting as an electron carrier in the mitochondrial electron transport chain while concurrently mitigating mitochondrial-derived oxidative stress. Beyond its primary function in ATP synthesis, CoQ10 exhibits potent neuroprotective properties by stabilizing mitochondrial membranes, reducing apoptotic signaling, and improving neuronal resilience against oxidative insults. Indeed, emerging evidence suggests that CoQ10 supplementation may ameliorate mitochondrial dysfunction and oxidative damage in nerve-degenerating conditions such as PD and AD [139]. Clinical trials have investigated its efficacy in delaying disease progression, though the findings to date remain inconclusive because of the variability in bioavailability and doubts about optimal dosing regimens [140].

In summary, recent studies suggest that while exogenous antioxidants offer potential neuroprotective benefits, their clinical efficacy in neurodegenerative diseases such as AD, PD, and ALS still requires further investigation [141]. Nonetheless, together, these antioxidants reduce the overall burden of oxidative stress and help preserve neuronal function, highlighting their therapeutic potential in the prevention and treatment of neurodegenerative diseases. Further work is clearly warranted to clarify the exact mechanisms and clinical efficacy of antioxidant-based interventions, paving the way for novel neuroprotective strategies.

## 9. Conclusions

Ochratoxins are toxic fungal metabolites present in a wide variety of foods and their by-products, including fruits, vegetables, cereals, meat, eggs, and dairy products, among others. The primary means of exposure to ochratoxins is oral and moreover, their ability to withstand elevated temperatures makes it difficult to eradicate them from the food supply. Among the different forms of ochratoxin, OTA is the most harmful; its metabolism generates hydroxylated derivatives (4-OH-OTA and 10-OH-OTA) and products conjugated with GSH as well as other derivates, which are all considered less toxic than OTA.

OTA has been described as nephrotoxic, hepatotoxic, teratogenic, and immunotoxic in several species and is neurotoxic and causes kidney and liver tumors both in animals and humans. In recent years, numerous research projects have aimed to determine the presence of OTA in various parts of the brain as well as its neurological effects. It has been shown that the main mechanisms of OTA action include the induction of oxidative stress, alteration of transcriptional regulation and cell signaling, inhibition of protein synthesis, interference with metabolic enzymes, cell cycle arrest, and the initiation of apoptosis. In this current review, we surveyed the cellular damage caused by OTA in different nervous system cell types. Thus, we described what is known about the neuroinflammatory effects of OTA in neurons, astrocytes, oligodendrocytes, and microglia.

Various in vitro and in vivo studies on OTA intake have demonstrated that this toxin can cross the BBB and generate damage at different levels of the brain in a dose-dependent manner. Perhaps because of the wide range of brain locations affected by OTA, its involvement in several different psychiatric disorders has also been demonstrated. However, despite these published data, further work will still be needed to completely explain the processes by which OTA crosses the BBB. In turn, here, we also discussed other studies suggesting that exogenous antioxidants may offer neuroprotective benefits. Nonetheless, their clinical efficacy in neurodegenerative diseases such as AD, PD, and ALS will still require further investigation. Together, these antioxidants reduce the overall burden of oxidative stress and help preserve neuronal function, thereby highlighting their therapeutic potential in the prevention and treatment of neurodegenerative diseases. Finally, more studies will be required to investigate ways of managing and reducing foods and animal feed contaminated with mycotoxins, safeguarding the health of people and animals, and lessening the associated financial losses.

## Data Availability

No new data were created or analyzed in this study.
